# Report of Severe Menorrhagia Following the Maximum Amount of Lamotrigine Overdose

**Published:** 2015

**Authors:** Farid Hajiali, Marjan Nassiri-Asl

**Affiliations:** a *School of Medicine, Qazvin University of Medical Sciences, Qazvin, Iran. *; b *Department of Pharmacology, Qazvin University of Medical Sciences, Qazvin, Iran.*

**Keywords:** Lamotrigine, Menorrhagia, Overdoses, Antiepilepic, Hypokalaemia

## Abstract

Lamotrigine is an antiepileptic drug used as a treatment for partial and generalised seizures as well as for bipolar disorder type I. Till date, very few cases of lamotrigine overdose have been reported. The spectra of clinical effects of lamotrigine in acute overdose are not well established. In severe cases of poisoning, serious effects such as coma, respiratory depression, recurrent seizures and intraventricular conduction disturbances have been noted. Here, we report a case of lamotrigine overdose in a 26-year-old divorcee with paradoxical seizure activity and coma. On admission, the patient had a reduced level of consciousness. Serum evaluation revealed high lamotrigine levels without any other aetiology for mental dysfunction. To the best of our knowledge, this is the first report to describe a patient overdosed with 40 g of lamotrigine alone, which is the highest amount of lamotrigine overdose reported so far. During hospitalisation, the patient’s haemoglobin level reduced from 12.9 to 7.7 g/dl and potassium level decreased repeatedly. More importantly, severe menorrhagia was noted. Following prompt supportive treatment with early intubation, along with the use of potassium chloride for hypokalaemia and administration of sodium bicarbonate, the patient’s conditions improved and she was discharged from the hospital after 13 days.

## Introduction

Lamotrigine [6-(2, 3-dichlorophenyl)-1, 2, 4-triazine-3, 5-diamine] is an antiepileptic drug, which has a broad spectrum of activities (1). It inhibits seizure activity by blocking glutamate release and depressing sodium channel neurotransmission (2). This drug is also used for the treatment of refractory bipolar disorder and trigeminal neuralgia (3), and has also been approved for long-term maintenance of bipolar mood disorder (4). It is commonly prescribed and, hence, it is important to understand its pharmacokinetics and the toxic effects of its accidental and intentional overdose. Lamotrigine is extensively metabolised in liver by N-glucuronidation (5). It is absorbed after oral administration rapidly and requires multiple dosing (2-3 times daily) for maintaining the therapeutic effect throughout the day (6, 7). Lamotrigine exhibits 55% protein binding and its metabolite, lamotrigine 2-N-glucuronide is eliminated by urinary excretion. A further 7% is renally excreted without any change (4, 8). However, its toxicokinetics is varied and its half-life has been reported to be between 10 and 110 h (9-11).The common adverse effects of lamotrigine include headache, insomnia, nausea, vomiting, anxiety, tremors (5), ataxia, nystagmus and vertigo, hypertonia, coma, convulsions, hypokalaemia and mental dysfunction (12). The major adverse drug reaction leading to lamotrigine discontinuation is skin rash, observed in 3-10% of the users (13). In a small percentage of patients, lamotrigine can cause serious adverse cutaneous reactions such as Stevens-Johnson syndrome (with median 50 mg lamotrigine in chronic use) and toxic epidermal necrolysis (with median 87.5 mg lamotrigine in chronic use) (5, 14). Haematological side effects of lamotrigine such as aplastic anaemia, bone marrow suppression and pancytopenia are relatively exceptional (with various doses of lamotrigine in chronic use) (15-17). One case of death due to complete heart block (with 4 g lamotrigine) (18) and one case of choreiform dyskinesia (with lamotrigine serum level of 63.9 μg/mL) (19) after lamotrigine overdose have been reported.

In this case report, we describe several side effects of this drug observed in a 26-year-old divorcee with intentional lamotrigine overdose (200 lamotrigine tablets of 200 mg). To our knowledge, this case report details the highest plasma level of lamotrigine reported so far, owing to the highest amount of lamotrigine overdose.

## Experimental

*Case report*

**Figure 1:**
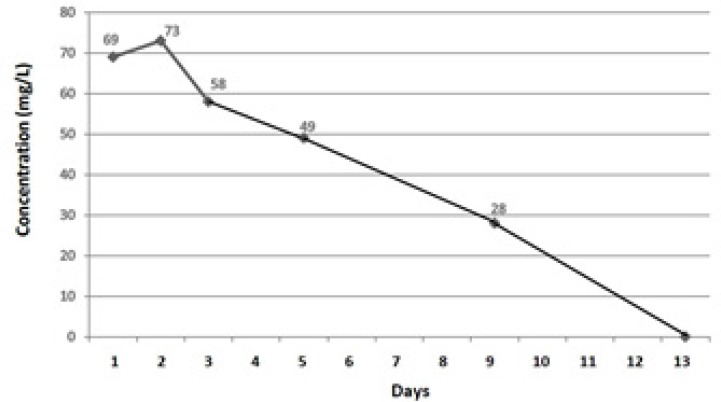
Lamotrigine serum levels during hospitalization.

A 26-year-old divorcee with a diagnosis of bipolar mood disorder, who acknowledged of attempting suicide about 40 min prior to being rushed to the hospital, was admitted to the Emergency Department (ED). Her family members informed that she had ingested 200 lamotrigine tablets of 200 mg (found from empty pill boxes). No history of vomiting after drug overdose was noted, and no medications were administered en route to the hospital. The patient was not on any other drug treatment and she denied taking any other medication at the time of this overdose. Serum evaluation revealed elevated lamotrigine levels without any other aetiology for mental dysfunction (Figure 1). The patient complained of weakness and lassitude. She was drowsy, agitated, tachycardic, lethargic, dehydrated and showed slurred speech, with cerebellar signs of nystagmus and ataxia. Within 20 min of admission to the ED, the patient asked for help beseechingly and then vomited immediately. After vomiting, the patient had generalised tonic-clonic seizure (full body ‘shaking’ movements lasting approximately 2 min). Then, her consciousness elapsed (with change in her Glasgow coma score (GCS) from 12/15 to 6–7/15) and therapeutic activities started. Her past medical history revealed that she did not have any systemic, organic or endocrine problems, except for a history of appendectomy that was done about 13 years ago and bipolar mood disorder for the previous10 years. Her past drug history showed that she had been on treatment with lithium and lamotrigine, but since 2 years before this suicidal attempt, only taking lamotrigine at a dose of 200 mg twice a day (maintenance treatment).

During initial examination on admission, the patient was agitated with a reduced conscious level (GCS of 12/15). Her oral temperature was 37.3°C and there was mild tachycardia and tachypnoea, with resting rates of 116 beats per minute and 32 breaths per minute, respectively. Her blood pressure was 185/95, but percutaneous oxygen saturation was 98%on room air. Her serum glucose level was 112 mg/dl when checked by using glucometer, and her pupils were 4 mm and reactive bilaterally. Papilloedema was absent, and corneal and vestibule-ocular reflexes were intact. The initial resting 12 lead electrocardiography (EKG) only showed sinus tachycardia (QRS interval, 96 ms) with localising or lateralising neurological signs were absent. Total blood count, urea, electrolyte and liver function tests were normal and within the reference ranges. Urinary toxicology screen was negative for amphetamines, methadone, cannabinoids, cocaine metabolites and opiates.

Initially, the patient had visual disturbance (blurred vision, diplopia), headache, dizziness and sweating, but skin and chest examination was normal with the exception of tachycardia. The patient complained of abdominal pain, but her abdomen was soft and non-tender and there were no signs of bladder distension. Although bowel sounds were noted, there was no evidence of bowel or bladder incontinence. Neurological examination revealed notable intact cranial nerves, horizontal nystagmus, decreased attention, inability to follow commands, inability to repeat the examiner’s phrases and markedly reduced language skills. The patient had spastic muscle tone with resistance to motion both with flexion and extension of all extremities. Deep tendon reflexes were noted to be 2 + brisk deep-tendon reflexes at biceps and patellar tendons without clonus, and Babinski reflex was absent. Following generalised tonic-clonic seizure after 20 min of presentation, the patient lost her consciousness (GCS of 6-7/15; she did not open her eyes, but responded to pain) and was intubated and ventilated. And after initial assessments on the first day, she was admitted to Intensive Care Unit (ICU) on the second day of admission. The lamotrigine serum levels observed in the patient are summarised in Figure 1.

*Management*

At the first day after intubation, an orogastric tube of the largest possible bore (36-French) was used for gastric lavage. Then, activated charcoal with sorbitol (1 g/kg of each) was administered into the stomach following gastric lavage. Intravenous fluid was administered because of the patient’s signs of dehydration and inability to accomplish enteral feeding (because of loss of consciousness). In every litre of normal saline, a bicarbonate sodium vial was added and administered to the patient for 2 days (used as a prophylactic treatment for deactivation of probable lamotrigine cardiotoxic effects). The patient’s consciousness level, blood pressure, heart rate and pulse oximetry along with ventilator were closely monitored. Folly catheter was fixed and the patient’s EKG did not exhibit any abnormal changes. The patient’s liver function tests were normal. Although phenobarbital was administered for the prevention of seizure recurrence, the patient had two seizures on the first and second days (two times of tonic-clonic seizure activity affecting all the four limbs, which was promptly terminated with intravenous diazepam (10 mg) administration. After the initial assessments, the patient was transferred to the ICU on the second day and was treated for 10 days. On the third day, the patient’s vital signs became normal and her GCS changed to 10 (she opened her eyes, produced incomprehensible sounds and obeyed commands); however, her GCS fluctuated between 7 and 12 till the eighth day. Finally, on the ninth day, the patient’s GCS changed to 14-15.

On the second day of admittance, the patient’s haemoglobin (Hb) level decreased from 12.9 (initial Hb) to 10.6 g/dl. However, on the third day, the patient’s Hb level was 7.7 g/dl and no apparent bleeding was noted. On primitive examination, we did not find any reason for this massive Hb drop. The patient’s physical examination and paraclinical tests were normal. Furthermore, urine analysis was normal, and occult blood tests revealed negative results without any evidence of haemolysis. Finally, on genitourinary examination, we found severe menorrhagia (but not proportional to the amount of Hb drop). Primitive examination of cervix and vagina with speculum test by gynaecologists did not reveal any abnormal findings. Subsequently, pregnancy test with abdominal and trans-vaginal ultrasonography was carried out. The patient’s serum level of beta human chorionic gonadotropin (B-HCG) was 0.1, ruling out any pregnancy-related problems. Furthermore, the patient’s abdominal and trans-vaginal ultrasonography was normal without any abnormality in ovaries and uterus. After supportive therapies and one packed cell transfusion, the patient’s Hb level changed to 11.8 g/dl, but decreased to 10.7 g/dl in less than a day. On receiving second packed cell transfusion, the patient’s Hb level reached 11.1g/dl, after which no Hb loss was noted. The total duration of menorrhagia was 2 days, which was finally stopped by intravenous administration of conjugated oestrogens.

The patient’s second complication was hypokalaemia and her potassium level repeatedly dropped for 5 days from the time of admittance, and the level was restored to the normal range by administering potassium chloride (KCl). The patient was extubated on the fourth day, but because of her low oxygen saturation and GCS fluctuation, she intubated again and finally extubated on the ninth day when her GCS was 14-15. The next day, the patient was transferred to the toxicology ward. Prior to the patient’s discharge, a psychiatric consultation was carried out to regulate her drugs (because of her bipolar mood disorder); however, the patient refused to cooperate with the psychiatrist and her family did not consent to continue with the consultation. Nevertheless, they agreed to consult with patient’s primary psychiatrist after discharge. The patient was discharged under healthy condition and strict recommendation was made to consult with her psychiatrist after 13 days from the day of hospitalisation.

## Results and Discussion

Very few cases of lamotrigine overdose have been reported. To our knowledge, our report is the first to describe a patient overdosed with 40 g of lamotrigine alone (200 lamotrigine tablets of 200 mg), that is the highest amount of lamotrigine overdose reported so far. The highest lamotrigine dose ever documented was 32 g (in mixed overdose with 11.5 g of pregabalin) that caused seizures and a reduced level of consciousness. (20). Our patient exhibited many of the most common clinical features of significant lamotrigine overdose mentioned earlier including ataxia, nystagmus and vertigo, hypertonia, coma, convulsions, hypokalaemia and mental dysfunction (12). The other signs included tachycardia and respiratory depression (21). Although an important observation in our patient was menorrhagia, we did not find any previous report of menorrhagia linked with lamotrigine in with literature. Our patient was not on any other medication, except lamotrigine. Her menstrual cycles were normal (in durations and intervals) and she had not been on any hormonal therapy. Her gynaecologic history was free from any problem, and she did not have any systemic, organic or endocrine problems. All of paraclinical tests were normal. She had not experienced any previous episodes of menorrhagia and became symptomatic only 1 day after a significant overdose of lamotrigine. In addition, her serum level of B-HCG was negative for pregnancy and abdominal and trans-vaginal ultrasonography were also normal without any abnormality in ovaries and uterus. Thus, it appeared that lamotrigine might be responsible for the menorrhagia observed in the patient.

Furthermore, despite the high amount of drug overdose, our patient did not show any adverse effects such as cardiac effects (e.g. widening of the QRS interval) (21), cutaneous reactions (2, 14) and acute pancreatitis (22), which have been reported for relatively lower toxic doses. Similar to our case, a recent case report documented high amountsof ingestion of lamotrigine (32 g) without any cardiac conduction abnormalities (20).

Clinical management of lamotrigine overdose generally consists of supportive care and correction of hydration status and any electrolyte imbalance. The conventional management of sodium channel blocking agent induced cardiotoxicity requires sodium bicarbonate administration. However, Sirianni *et al .*, suggested that lipid infusion in the treated case of refractory cardiovascular collapse due to overdose of lamotrigine and bupropion could result in reducing the toxicity of lamotrigine by bounding to it and help to its excretion (23).

Owing to the corroborative serum lamotrigine concentrations, the lack of co-ingested drugs and the reasons mentioned earlier, we suggest that significant lamotrigine overdose might have caused menorrhagia in our patient. In addition, other side effects such as agitation, ataxia, nystagmus and vertigo, hypertonia, coma, possible seizures, possible haemopoietic suppression, sinus tachycardia and respiratory depression were also evident. The populations prescribed lamotrigine are at a high risk of overdose. A third of individuals with bipolar disorder admit to at least one suicide attempt.

## Conclusion

The purpose of this case report was to suggest that physicians should pay more attention to the potential of lamotrigine to cause menorrhagia, and should always consider this side effect (along with other side effects) and subtilise prescription of lamotrigine for any patients and at any doses, especially for patients with a previous history of drug or substance abuse and at high doses. (24)
